# Phase Transformations and Mechanical Properties in In–Bi–Sn Alloys as a Result of Low-Temperature Storage

**DOI:** 10.3390/ma17153669

**Published:** 2024-07-25

**Authors:** Jiye Zhou, Xin Fu Tan, Stuart D. McDonald, Kazuhiro Nogita

**Affiliations:** Nihon Superior Centre for the Manufacture of Electronic Materials (NS CMEM), School of Mechanical and Mining Engineering, The University of Queensland, St. Lucia, QLD 4072, Australia; jiye.zhou@uq.edu.au (J.Z.); xin.tan@uq.edu.au (X.F.T.);

**Keywords:** low-temperature environment, mechanical property, ternary alloys, phase transformation

## Abstract

The In–Bi–Sn low-temperature solder alloys are regarded as potential candidates for cryogenic and space exploration applications. This study investigates the variations in the mechanical properties and microstructures of two different compositions: In15wt%Bi35wt%Sn and In30wt%Bi20wt%Sn, after exposure to a low-temperature environment (−20 °C) for 10 months. An increase in the ultimate tensile strength was observed across all the tested samples and a decrease in elongation to failure was observed in In30wt%Bi20wt%Sn. Changes in the microstructure were identified through scanning electron microscopy (SEM) and electron backscatter diffraction (EBSD). The impact of this low-temperature environment is described, considering the varying proportions and compositions of the three phases (BiIn_2_(Sn), γ-InSn_4_(Bi), and β-In_3_Sn(Bi)) present within the alloys and their contribution to the mechanical properties.

## 1. Introduction

The growing interest in space exploration and other cryogenic applications like quantum computers and superconducting devices is driving the need to develop solder alloys for the assembly of electronics for deployment in extreme conditions. For example, the significant temperature shifts on Mars, which can range from +20 °C to −90 °C, require solders with stable properties under variable temperatures for long-term use [1,2,3]. For these applications, solder alloys must withstand long-term use in extreme temperatures while maintaining the structural integrity of joints with Cu-based substrates. This requires providing stable mechanical bonding of the joints and sufficient ductility to mitigate stresses between printed circuit boards (PCBs) and components in the service environment [4,5]. Furthermore, the ease of electronics assembly, particularly in cryogenic environments, necessitates lower melting points and good wettability at low temperatures [5]. Compared to tin-based lead-free solder alloys, indium-based solder alloys are known for their low melting temperatures and their capacity to remain ductile even at very low temperatures [4,5,6,7]. Research into binary indium–tin (In–Sn) alloy compositions has shown that the differences in mechanical behaviours are closely aligned with the proportions of the ductile β-In_3_Sn phase and the relatively less ductile but stronger γ-InSn_4_ phase [6]. Compared to the In–Sn binary alloys, indium–bismuth–tin (In–Bi–Sn) ternary alloys can have a lower melting point of around 59 °C and offer a versatile solution offering energy savings, cost reduction through lower indium use, and better ductility without sacrificing strength. It has been reported that the presence of BiIn_2_(Sn) and β-In_3_Sn(Bi) in In–Bi–Sn alloys has been linked to exceptional ductility, with certain compositions achieving remarkable elongation, which may improve the reliability of solder alloys operating in cryogenic conditions [7,8,9,10].

Ensuring the durability of solder alloys in extreme conditions over an extended period presents a significant challenge. Due to the polymorphism in Sn, it has been observed that tin-based solders can shift from being ductile to being brittle when exposed to cryogenic temperatures, which is attributed to an increase in slip activation stress with decreasing temperature [5,11]. Additionally, research focusing on the cryogenic mechanical behaviours of indium-based alloys has shown that pure indium can reach a 37% elongation compared to the 34% detected in eutectic In–48Sn (all compositions are in wt% unless specified otherwise) and 22% in eutectic In–32.5Bi–16.5Sn when tested at −20 °C with a strain rate of 2.65 mm/min [12]. Madhuri et al. reported minimal differences in elongation between tests conducted at room temperature and at −196 °C for pure indium and In–Bi alloys, yet In–Bi binary alloys demonstrated a significant (over 750% in In–51wt%Bi and 920% in In–34wt%Bi)) increase in ultimate tensile strength at cryogenic temperatures, indicating that the hardening effect was strongly influenced by temperature in this system [12]. Additionally, it was found that Cu/In–34Bi–20Sn/Cu solder joints possess excellent shear strength at −196 °C and maintain this strength even after undergoing thermal cycling [3].

Our recent investigations have indicated a notable reduction in the elongation of In–Bi–Sn solder alloys at cryogenic temperatures, with a corresponding increase in the ultimate tensile strength [3,10,12]. It has been shown [3,10,12] that the In15wt%Bi35wt%Sn alloy, which has tetragonal β-In_3_Sn(Bi) as the main phase, exhibits more stable mechanical properties under temperature variations, while for the near-eutectic In30wt%Bi20wt%Sn sample, which has a hexagonal BiIn_2_(Sn) as the main phase and a large proportion of hexagonal InSn_4_(Bi) phase, the elongation dropped from over 250% at room temperature to approximately 7% at −90 °C. These changes are associated with the suppression of thermally activated deformation mechanisms at lower temperatures and the formation of BiIn_2_(Sn) and γ-InSn_4_(Bi) phases, which have limited slip systems [10]. Alloys containing a significant amount of these phases at cryogenic temperatures experienced more pronounced decreases in elongation, indicating that the proportion of the three phases is critical. The transformation of phases in this ternary system is time–temperature-dependent [13,14], suggesting that being exposed to lower temperatures can cause phase transformations, potentially compromising the reliability of the solder alloys [10].

As the operating environment is critical and may affect the reliability of the solder by altering its microstructure and deformation mechanisms over an extended period, it is essential to evaluate which phase maintains the most stable mechanical properties after long-term exposure to harsh environments, thereby improving the overall performance of the solders. Furthermore, most studies on indium-based solder alloys focus on the properties of as-cast samples rather than the impact of the service environment on their properties. In this work, we assess the mechanical properties of these two In–Bi–Sn solder alloys with different volume fractions of each phase after long-term storage at −20 °C. We discuss the findings in the context of microstructural changes and fracture morphologies to evaluate the reliability of In–Bi–Sn solder alloys for lower-temperature applications, particularly at temperatures nearing −20 °C.

## 2. Materials and Methods

To obtain tensile bars for testing, pure indium (99.995%) ingots (supplied by Nihon Superior, Osaka, Japan), tin (99.99%) ingots, and bismuth (99.9%) ingots (supplied by Northern Smelters, Woodridge, QLD, Australia) were alloyed to obtain two different compositions (shown in Table 1): In–15wt%Bi–35wt%Sn (S1) and In–30wt%Bi–20wt%Sn (S2). These alloys were chosen as they contain different volume fractions of β, γ, and BiIn_2_ phases in the microstructure and have demonstrated exceptional ductility in as-cast samples from our previous work [10]. Weighed ingots were melted in boron–nitride-coated clay–graphite crucibles in an electric resistance furnace at temperatures of 220 °C above their equilibrium melting point for one hour, and no protective atmosphere was used in the furnace. Any dross formation on the surface of the liquid metal was mechanically removed, and stirring was performed every fifteen minutes to achieve a homogenous melt and prevent oxidation from impacting the results. Then, alloys were cast into a boron–nitride coated aluminium mould with a cavity that produced tensile bars compliant with the ASTM E8/ standard [15] and then allowed to air cool. The cylindrical bars had a parallel length of 38mm and a reduced nominal diameter of 6mm, which are the same as those used in our previous studies [6,10].

The tensile testing was performed in an Instron (model 5584) machine with a 1 kN load cell for both compositions, and the crosshead displacement rate was fixed at 1.8 mm/min (~0.0008 s^−1^). A video extensometer was used to measure the strain during testing. A 30 mm gauge length was used for the video extensometer, and the diameter of the reduced section of the tensile bar was 6 mm. All the cast tensile samples were placed in a freezer with the temperature calibrated at −20 °C for 10 months before testing. For each composition, casting defects or surface defects were observed on casted tensile bars, which were induced by turbulent pouring or uneven cooling rates. The presence of defects such as porosity or inclusions is always a possibility in cast alloys and these issues are well recognised to be associated with reduced elongation [16]. In this work, three tensile bars were tested and the average tensile data was calculated from the two samples that had the largest elongation and then compared to the as-cast samples from our previous study [10].

Comparative microstructural analysis was conducted on the polished surfaces of In–15wt%Bi–35wt%Sn and In–30wt%Bi–20wt%Sn block samples before and after freezing at −20 °C for 10 months. Additionally, to analyse the microstructure after tensile testing, the materials in the vicinity of the fracture were cross-sectioned, mounted in resin, and prepared using the standard polishing procedure. For all the samples, the preparation procedure involved grinding with silicon carbide papers to a 4000-grit finish, followed by polishing with a 1 μm diamond suspension and a 0.04 μm colloidal silica suspension for two minutes each. Ice water was used as a lubricant to prevent any heat generation during the grinding and polishing process, which may induce phase transformations above room temperature. The microstructure images of the prepared samples were captured using a scanning electron microscope (SEM) (Hitachi TM3030, Tokyo, Japan) under backscatter electron (BSE) mode at a 15 kV accelerating voltage, and energy dispersive X-ray spectroscopy (EDX) analysis for elemental mapping was carried out using Bruker Quantax 70 software. The proportion of each phase in the tested samples was further analysed using ImageJ software (version 1.54). The electron backscatter diffraction (EBSD) analysis of the fracture surface was performed at a voltage of 20 kV and an 11 nA probe current on a JOEL JSM-7800F (Tokyo, Japan) SEM equipped with an Oxford HKL EBSD system.

## 3. Results and Discussion

### 3.1. Microstructural Analysis

**Figure 1:**
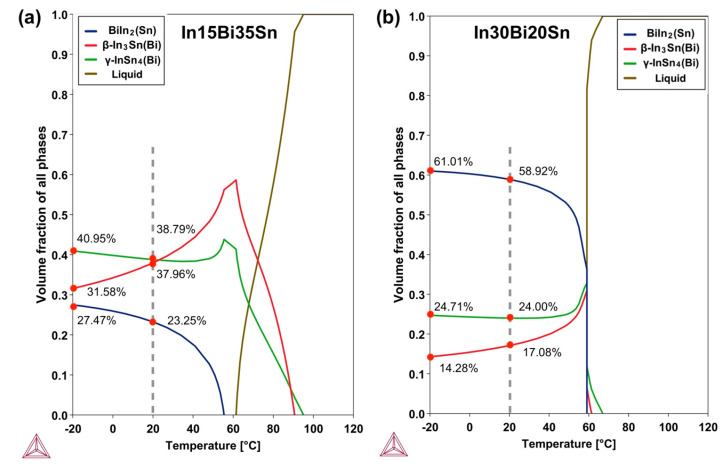
Equilibrium volume fraction of phases between −20 and 120 °C in In–Bi–Sn alloys calculated based on the Thermo-Calc 2024 [17] database (TCSLD4: Solder Alloy v4.1). (**a**) In15Bi35Sn; (**b**) In30Bi20Sn.

**Figure 2:**
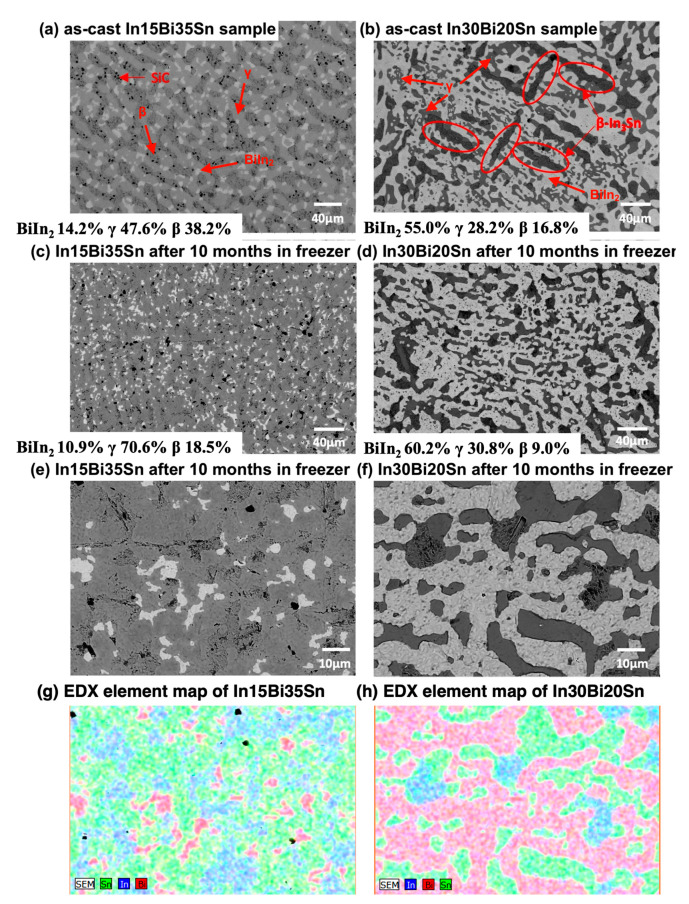
Microstructure of (**a**) as-cast In15Bi35Sn; (**b**) as-cast In30Bi20Sn; (**c**) In15Bi35Sn sample after freezing at −20 °C for 10 months; (**d**) In30Bi20Sn sample after freezing at −20 °C for 10 months; (**e**) high mag. image of In15Bi35Sn; (**f**) high mag. image of In30Bi20Sn; (**g**) EDX element map of In15Bi35Sn; (**h**) EDX element map of In30Bi20Sn.

Figure 1 shows the CALPHAD (Thermo-Calc 2024 [17]) generated equilibrium change in the volume fraction of each phase during cooling for both experimental alloys. There was a significant difference between the as-cast S1 and S2 samples in terms of the proportion of different phases present. Based on Thermo-Calc predictions, In15Bi35Sn had a similar proportion of β and γ at room temperature, which was around 38%, and a smaller amount of BiIn_2_(Sn) (23%). This was consistent with the microstructure of the as-cast sample captured by SEM (see Figure 2a). For the In30Bi20Sn sample, the prediction of the volume fraction of each phase at 20 °C and −20 °C agreed with the SEM results (see Figure 2); the microstructure primarily consisted of a BiIn_2_(Sn) matrix, which was close to 60%, with a considerable portion of the γ phase (around 24%) and a small amount of dispersed β phase present (less than 17%). As shown in Figure 1, both samples underwent a phase transformation from β to BiIn_2_(Sn) and γ when the temperature decreased to −20 °C, resulting in an approximate 6% and 3% decrease in the proportion of β for In15Bi35Sn and In30Bi20Sn, respectively. In comparison, in the analysis conducted using ImageJ (displayed in Figure 2), the proportion of the BiIn_2_(Sn) and γ phases slightly increased after storage in the freezer, while the proportion of the β phase decreased accordingly. This trend was consistent with the predictions made by Thermo-Calc during cooling.

As shown in Figure 2a,b, compared to the irregular and island-like γ grains in the as-cast In–Sn binary alloys [6], the as-cast In15Bi35Sn exhibited a more uniform lamellar microstructure consisting of β (dark) and γ (grey) phases with some BiIn_2_(Sn) precipitates interspersed between them. Based on the Thermo-Calc prediction, the In15Bi35Sn sample had a primary γ phase formed during cooling followed by the solidification of a large amount of β + γ phases, which underwent a phase transformation to BiIn_2_(Sn) when the temperature dropped below 60 °C. While In30Bi20Sn, a near-eutectic composition, had a more irregular lamellar eutectic microstructure consisting of β, γ, and BiIn_2_(Sn) [10,13,14]. As shown in Figure 1b, a small amount of proeutectic β + γ phases formed during solidification, and the figure also shows the presence of both coarse and fine γ and β grains depending on parameters such as the temperature gradient, the cooling rate, or the solidification characteristics of each phase [18]. For both alloys, the microstructure of the In–Bi–Sn eutectic was comparable to that of the Sn–Bi eutectic with distinct phases arranged in a lamellar pattern [18,19].

Compared to the as-cast sample, it was observed that the microstructure of the sample stored in the freezer for 10 months contained some fine BiIn_2_(Sn) precipitates and an increased volume of the γ phase, with a less uniform phase distribution. The proportions of the three phases in the microstructure were analysed using ImageJ software (version 1.54). For the as-cast sample in Figure 2a, the γ phase (47.6%) was the main phase, with a close proportion of β (38.2%) and a small amount of BiIn_2_(Sn) (14.2%) in the microstructure. Furthermore, the EDX mapping shown in Figure 2g and the ImageJ analysis of Figure 2c revealed an increase in the proportion of the γ phase (70.6%) in In15Bi35Sn and a decrease in the BiIn_2_(Sn) (10.9%) and β (18.5%) phases after being stored in the freezer for 10 months. The increase in the proportion of the γ phase in the sample stored in the freezer can be attributed to either the inhomogeneity of the sample or phase transformations and nonequilibrium microstructures caused by experiment or sample preparation. It is worth noting that due to the softness of the β phase, it exhibited an inferior surface finish compared to the other two phases (shown in Figure 2a–f), and some SiC particles were embedded in the β phase during grinding.

For In30Bi20Sn (S2), there was minor difference between the microstructure of the as-cast sample and the sample stored in the freezer for 10 months, except for a slight increase in the proportion of BiIn_2_(Sn) and γ phases and a slight decrease in the proportion of β. Dendrites, which are associated with the solidification of the β and γ phases prior to the eutectic reaction, were observed in the as-cast microstructure (Figure 2b). Additionally, fine γ grains were observed in the microstructure for the In30Bi20Sn sample both before and after storage in the freezer. For both conditions, the eutectic β phase was located adjacent to the large γ phase. Compared to In15Bi35Sn, the proportions of the three phases in the microstructure of the In30Bi20Sn samples (Figure 2b,d) showed better agreement with the Thermo-Calc predictions.

### 3.2. Mechanical Properties

**Figure 3:**
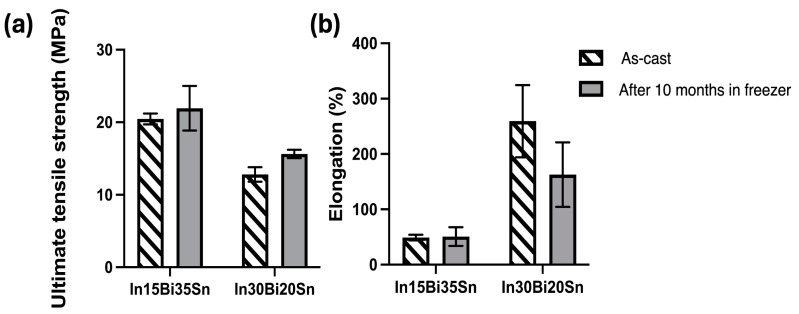
(**a**) Ultimate tensile strength of as-cast samples and samples after freezing at −20 °C for 10 months; (**b**) Elongation of as-cast samples and samples after freezing at −20 °C for 10 months.

The comparison of the mechanical properties between the samples before and after freezing at −20 °C is displayed in Figure 3, and the data for the individual samples are listed in Table A1. The relatively large standard deviation (SD) for the elongation, especially in In30Bi20Sn, may be associated with material inhomogeneity, defects caused by casting, or local volume changes caused by reprecipitation after freezing. The average UTS and elongation of as-cast samples from our previous study were calculated from three samples that had the largest elongation at each composition [10]. Due to the larger amount of γ phase in the microstructure, all the In15Bi35Sn samples had a higher UTS than In30Bi20Sn. Compared to an as-cast near-eutectic In–50Sn alloy [6], which contains approximately 45% γ phase and 55% β phase at 25 °C, the ultimate tensile strength (UTS) of the In15Bi35Sn alloy was slightly higher, at around 20 MPa compared to 16 MPa. Meanwhile, the In15Bi35Sn alloy exhibited higher elongation, with 50.7% compared to 37.8% under the same testing conditions. The elongation of the tested In–Bi–Sn samples was also higher compared to well-accepted solder alloys such as Sn3Ag0.5Cu, which has around 43% elongation, as tested under the same strain rate in our previous study [20]. As shown in Figure 3, for both compositions, the samples after freezing at −20 °C showed an increase in the average UTS, especially in the In30Bi20Sn, which showed a 21% increase from 12.81 MPa to 15.6 MPa. The γ and BiIn_2_(Sn) phases have a hexagonal crystal structure (P_6_/mmm and P_63_/mmc), which has fewer slip systems and is, therefore, more brittle, while the β phase with a body-cantered tetragonal (I_4_/mmm) crystal structure has more slip systems and is more ductile [6,10]. The increasing UTS can be attributed to a slight increase in the volume fraction of the more brittle BiIn_2_(Sn) and γ phases and a decrease in the volume fraction of the ductile β phase when subjected to lower temperatures for both compositions (refer to Figure 1), and such a microstructural change is difficult to reverse in the short time that the sample experienced room temperature prior to tensile testing.

**Figure 4:**
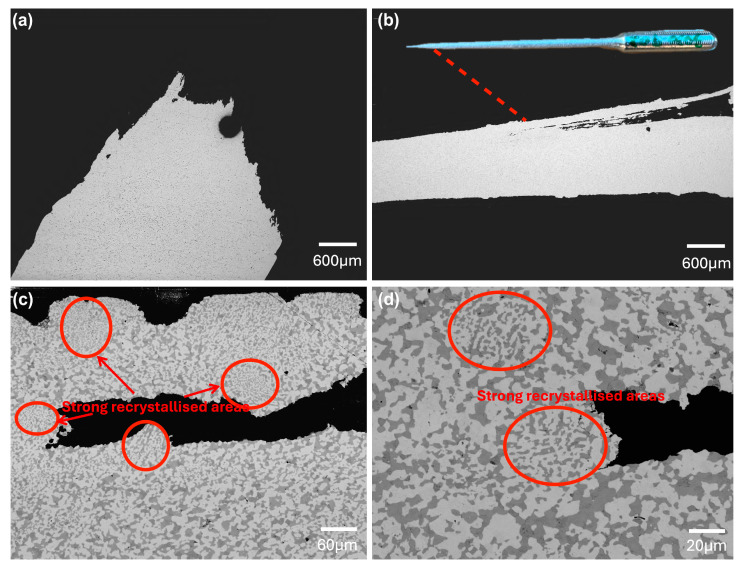
Representative cross-section microstructures of the fracture region in tested samples after freezing at −20 °C for 10 months. (**a**) In15Bi35Sn; (**b**) In30Bi20Sn; (**c**) Void nucleation in In30Bi20Sn with strong dynamic recrystallised areas around it labelled by red circles; (**d**) Microstructure adjacent to the void within In30Bi20Sn at high magnification.

In terms of elongation, the In15Bi35Sn (S1) samples showed a consistent value of approximately 50% elongation both before and after 10 months of storage in the freezer. However, despite the exceptional ductility, the elongation of In30Bi20Sn dramatically decreased after 10 months from 259.4% in the as-cast samples [10] to 162.9%. As shown in Figure 4a, the fracture surface of the In15Bi35Sn alloy showed a slightly narrowed neck, characteristic of a ductile failure, where significant plastic deformation occurs before the material ultimately breaks. This type of failure is often associated with the presence of dimples close to or on the fracture surface, signifying microvoid coalescence during tensile testing [21]. In30Bi20Sn (Figure 4b) showed an elongated cone shape, which was necked down to a fine point after tensile testing, indicating a more pronounced plastic deformation. Figure 4c,d shows a pore in the tested In30Bi20Sn tensile bar and the microstructure adjacent to this pore, and this pore nucleation is associated with the failure of this alloy. During the tensile testing, there were several nucleations of porosities occurring in the microstructure simultaneously. At the same time, the nucleation of new grains and recrystallisation occurred at the grain boundaries where voids started forming, allowing the alloy to achieve exceptional ductility [10,22]. As labelled in Figure 4c,d, there were several areas that exhibited much stronger dynamic recrystallisation and smaller grain sizes, which were susceptible to pore nucleation and were subsequently filled by new grains during tensile testing. Additionally, despite some small areas at the interface with the large pore showing some recrystallisation, most areas at the interface were not, indicating a weak dynamic recrystallisation behaviour. Therefore, when the speed of dynamic recrystallisation cannot accommodate the nucleation and coalescence of porosity, these voids accumulate together and form a big pore (the one shown in Figure 4b–d) until fracture occurs.

The tensile testing results showed a decrease in elongation in In30Bi20Sn (S2) after storage in the freezer for 10 months as the thermal-activated deformation mechanisms were weakened. Our previous study highlighted the significant influence of thermal-activated deformation mechanisms in In30Bi20Sn composition and their great contribution to the overall strain [10]. For the In–Bi–Sn alloy system, which has a high homologous temperature, atoms can easily migrate through the crystal lattice or along grain boundaries even at room temperature, facilitating creep, boundary sliding, and dynamic recrystallisation. Consequently, diffusional creep, which is a time-dependent and temperature-dependent deformation process, occurs primarily through these diffusion mechanisms, thereby facilitating plastic deformation [23,24,25]. In contrast, for samples stored in the freezer for a longer time, the thermal-activated deformation was weakened by three factors. First, the hardening effect associated with the phase transformation from β-In_3_Sn(Bi) to BiIn_2_ and γ-InSn_4_(Bi) contributed to the increase in the UTS, as BiIn_2_ and γ-InSn_4_(Bi) are both harder than the β-In_3_Sn(Bi) due to the limited slip systems in their hexagonal structures. However, as the movement of dislocations became more difficult, the elongation decreased accordingly. Secondly, as the diffusion was most active between the β phase and the other two phases, a decrease in the amount of β-In_3_Sn(Bi) resulted in reduced sliding and boundary diffusion occurring at the β-BiIn_2_(Sn) or β-γ interfaces. Finally, the precipitation of BiIn_2_ or γ-InSn_4_(Bi) may have led to a less uniform microstructure, which is unfavourable for achieving superplasticity [26]. In contrast, the In15Bi35Sn sample relied less on diffusion-related deformation mechanisms and more on the ductility of the β phase itself [10].

**Figure 5:**
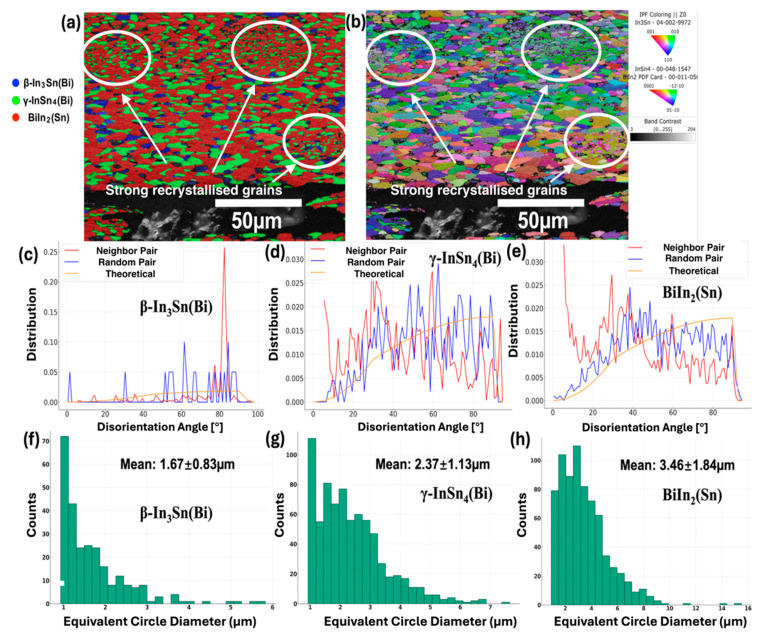
EBSD maps of In30Bi20Sn after freezing at −20 °C for 10 months. (**a**) Phase map of cross-section of In30Bi20Sn tensile bar after tensile testing in the vicinity of the fracture and strong dynamic recrystallised grains; (**b**) Corresponding inverse pole figure (IPF); (**c**–**e**) Grain disorientation in β–In_3_Sn(Bi), γ–InSn_4_(Bi), and BiIn_2_(Sn) respectively; (**f**–**h**) Grain size distribution in β–In_3_Sn(Bi), γ–InSn_4_(Bi), and BiIn_2_(Sn), respectively.

Figure 5 shows the EBSD analysis conducted on the cross-section surface adjacent to the fracture point of a tested In30Bi20Sn tensile bar (an area within Figure 4b). The map was captured for grains surrounding a pore, which is associated with a ductile failure mode. As shown in Figure 5a,b, grains in the microstructure were elongated along the direction of applied tensile force. Additionally, finer grains and random orientation were observed compared to the bulk sample for the same alloy (Figure 2d).

The EBSD data indicated a volume fraction of each phase as follows: 7.6% of β–In_3_Sn(Bi), 28.7% of γ–InSn_4_(Bi), and 64.7% of BiIn_2_(Sn), which were close to the Thermo-Calc predictions of 14%, 25%, and 61%, respectively, that are shown in Figure 1b and the SEM image in Figure 2d. The lower volume fraction of β may be attributed to difficulty indexing, as it is the softest phase in the microstructure and is easily contaminated during polishing. By combining Figure 4 and Figure 5a,b, the whole elongated microstructure of In30Bi20Sn showed dynamic recrystallisation to some extent after tensile testing and several strong recrystallised areas were observed. By identifying the individual grain boundaries within each phase, the mean grain size was approximately calculated (as shown in Figure 5f–h) as 1.67 µm, 2.37 µm, and 3.46 µm for β–In_3_Sn(Bi), γ–InSn_4_(Bi), and BiIn_2_(Sn), respectively. The average grain size of the three phases was relatively small and exhibited disorientation indicative of strong dynamic recrystallisation in all three phases during tensile testing. In contrast, the polished bulk sample showed an average grain size of 26.97 µm in the BiIn_2_(Sn) phase [10], further supporting the occurrence of significant recrystallisation during tensile testing.

As seen on the fracture surface shown in Figure 4 and Figure 5, several locations underwent more severe dynamic recrystallisation during tensile testing, showing fine grains, which were mostly recrystallised γ–InSn_4_(Bi) and BiIn_2_(Sn). Notably, these recrystallised grains showed the same orientation within a given area and a similar orientation between different spots. This indicates that the preferred orientation along the <$\bar{1}2\bar{1}0$> and <01$\bar{1}$0> directions for the strong recrystallised BiIn_2_(Sn) and γ grains, as observed on the polished surface, originate from growth selection of energetically favourable directions [27], regardless of the extent of deformation. Furthermore, the highly directional recrystallisation in these spots may be associated with uneven distribution of plastic deformation due to the geometric or crystallographic instabilities in the local area and localised shear deformation associated with the nucleation of voids during the tensile test. As shown in Figure 5d,e, despite these uniform, strongly recrystallised spots, the γ–InSn_4_(Bi) and BiIn_2_(Sn) crystals exhibited a random distribution of the disorientation angles in the recrystallised microstructure. Therefore, the highly directional and strong recrystallisation may only be associated with the area that had porosity or anisotropic stress distribution.

There is some evidence that shows the increase in the UTS and the decrease in elongation were correlated to the degree of grain disorientation. The majority of neighbour grain boundaries in the β phase had a high disorientation angle of around 85° (shown in Figure 5c), and these were likely to contribute to pinning, therefore leading to a stronger hardening effect in these regions. However, these effects may not have significantly contributed to the overall mechanical properties, considering that these areas were limited within the microstructure.

The precipitation of BiIn_2_(Sn) and the γ phases strengthened the alloy while decreasing the ductility by acting as barriers to dislocation movement, thereby increasing the creep resistance.

## 4. Conclusions

Combining the discussion and all experimental data in this paper, several conclusions can be drawn as follows:

- The ultimate tensile strength (UTS) of In–Bi–Sn solder alloys increased after storing at −20 °C for extended periods for both compositions. In30Bi20Sn showed a decrease in elongation, whereas In15Bi35Sn, which contains a larger proportion of the ductile β phase, exhibited minimal change in elongation. The precipitation of the more brittle hexagonal BiIn_2_(Sn) and γ phases is the primary reason for the decreased elongation, as these phases strengthen the alloy while reducing ductility by acting as barriers to dislocation movement, thereby increasing the creep resistance of the alloy.
- Notable changes in the proportion of phases in the microstructure, as predicted by Thermo-Calc, were verified by SEM results. Compared to the as-cast samples, phase transformations from β to γ + BiIn_2_(Sn) were observed in the original β phase for both samples after freezing at −20 °C for extended periods.
- Phase transformations and hardening due to the reprecipitation of phases have a more significant impact on the mechanical properties of alloys with a high amount of γ or BiIn_2_(Sn) phases after solidification while having less impact on alloys with a high amount of the ductile β phase. Despite the phase transformations, the ductile fracture mode of the tested alloys remained consistent with that of the as-cast samples, and strong recrystallization was observed.
- For operating environments ranging from room temperature to below −100 °C, future research should explore other indium-based solder alloy compositions, particularly those with a high amount of the ductile β phase, which are shown to have more stable mechanical properties under temperature variations. Additionally, conducting long-term testing under thermal cycling at different temperature ranges on In–Bi–Sn/Cu solder joints could provide more insights into the reliability and performance of these alloys in real-world applications.

## Figures and Tables

**Table 1:** In–Bi–Sn alloys studied in this paper.

Test Sample	S1	S2
Composition	In–15wt%Bi–35wt%Sn	In–30wt%Bi–20wt%Sn

## Data Availability

The data presented in this study are openly available in the manuscript.

## Appendix A

**Table A1:** Ultimate tensile strength and elongation of tested samples in this study. (SD refers to the standard deviation of the elongation measurements).

Test Sample	Strain Rate	UTS (MPa)	Elongation (%)	SD (%)
In–15wt%Bi–35wt%Sn (S1)	1.8 mm/min	24.11	38.8	16.9
		19.77	62.75	
In–30wt%Bi–20wt%Sn (S2)		16.04	204.1	58.2
		15.23	121.6	
